# Predictors of Stress in College Students

**DOI:** 10.3389/fpsyg.2017.00019

**Published:** 2017-01-25

**Authors:** Dalia Saleh, Nathalie Camart, Lucia Romo

**Affiliations:** ^1^EA4430 CLIPSYD, UFR SPSE, Paris Nanterre UniversityNanterre, France; ^2^Counseling Psychology, Tishreen UniversityLatakia, Syria

**Keywords:** college students, perceived stress, optimism, self-efficacy, psychological distress, self-esteem

## Abstract

University students often face different stressful situations and preoccupations: the first contact with the university, the freedom of schedule organization, the selection of their master's degree, very selective fields, etc. The purpose of this study is to evaluate a model of vulnerability to stress in French college students. Stress factors were evaluated by a battery of six scales that was accessible online during 3 months. A total of 483 students, aged between 18 and 24 years (Mean = 20.23, standard deviation = 1.99), was included in the study. The results showed that 72.9, 86.3, and 79.3% of them were suffering from psychological distress, anxiety and depressive symptoms, respectively. More than half the sample was also suffering from low self-esteem (57.6%), little optimism (56.7%), and a low sense of self-efficacy (62.7%). Regression analyses revealed that life satisfaction, self-esteem, optimism, self-efficacy and psychological distress were the most important predictors of stress. These findings allow us to better understand stress-vulnerability factors in students and drive us to substantially consider them in prevention programs.

## Introduction

Many studies highlighted mental health issues in young adult, especially during their studying years at university (Blanco et al., 2008; Milojevich and Lukowski, 2016).

According to health surveys, young people from 12 to 25 years old suffer from an insufficient level of psychological health (Grebot and Barumandzadeh, 2005; Windfuhr et al., 2008; Thapar et al., 2012). Some studies also show that, compared to individuals of the same age (Roberts et al., 1999; Adlaf et al., 2005; Boujut et al., 2009) and, in general, to any other population (Nerdrum et al., 2006; Blanco et al., 2008; Walsh et al., 2010; Moreira and Telzer, 2015), students have more psychological problems.

Students' psychological discomfort is reflected in several ways including depression, anxiety, stress, and sleeping disorders (Lejoyeux et al., 2011; Schraml et al., 2011; Boulard et al., 2012; Nyer et al., 2013; Petrov et al., 2014; Feld and Shusterman, 2015; Milojevich and Lukowski, 2016). This discomfort has been the subject of many investigations. In fact, depression is common in students from 15 to 24 years olds (Lafay et al., 2003). According to a French study (Boujut et al., 2009), 27, 18, and 3% of college students suffer from mild, moderate and severe depression, respectively. Anxiety (Neveu et al., 2010) and feeling overwhelmed are actually quite typical of college students, including those who succeed (Lassarre et al., 2003). More than 83% of students from the University of Lodz suffer from fatigue (Maniecka-Bryła et al., 2005). In addition, according to two French studies, 15% of students had suicidal thoughts (Lafay et al., 2003) while 3% had a suicidal tendency (Boujut et al., 2009). It seems that suicidal thoughts are more prevalent, during the past 12 months, in students than other young people (Grémy et al., 2002). Furthermore, another study found that 60% of first-year students of a business school (Ecole Supérieure de Commerce) had significant levels of psychological distress and low self-esteem (Strenna et al., 2009). Their coping strategies were principally based on withdrawal (Strenna et al., 2009). Humphris et al. (2002) found that more than 30% of European dental students reported significant psychological distress and 22% reported a high level of emotional exhaustion.

These mental health issues among students are of growing concern (Castillo and Schwartz, 2013; Milojevich and Lukowski, 2016). It should be noted that in most cases, men report being less anxious and depressed (Castillo and Schwartz, 2013) and having less suicidal thoughts than women (Dusser et al., 2009).

This poor psychological well-being is sometimes associated with physical disorders (Graziani et al., 2001). It could also be associated with the broader concept of “stress,” that involves all aspects of life's difficulties, including psychological discomfort. Each student deals with the same stress differently (Boujut, 2007). A French study showed that 79% of students reported being stressed (Vandentorren et al., 2005).

On the other hand, other studies focused on the factors that were linked to these issues in students and found that neuroticism, which is the general tendency to experience unpleasant or negative emotions, could be a stress predictor in students (Vollrath, 2000). Nonetheless, low self-esteem was found to be the strongest predictor of stress symptoms (Han, 2005). Strenna et al. (2009) found a link between low self-esteem and anxiety and depression in students.

Little research is currently being conducted on the psychological health of college French students (Boujut, 2007; Strenna et al., 2009). Ongoing research has mainly been carried out as part of students' health insurance surveys (Boujut et al., 2009). The main purpose of this study is to determine the role of some factors (gender, age, year of studies, formation and research units (UFR), self-esteem, optimism, self-efficacy and psychological distress, including somatic symptoms, anxiety, insomnia, social dysfunction and severe depression) linked to the presence of perceived stress in students, after evaluating different aspects of mental health in college students. This could help to better evaluate and understand the psychological malaise of French college students.

## Methods

### Data collection procedure

Online data collection was conducted between February and May 2014, using a battery of questionnaires posted on Googles docs. Information regarding this survey was distributed to all registered students at the University Paris Ouest Nanterre La Defense and other universities in the Parisian area.

### Ethics statement

The study protocol was approved by the ethics committee of the Psychological Science and Learning Science department at the University of Paris Ouest Nanterre La Défense, UFR SPE (Department of Psychology and Education) and by the CNIL (Commission nationale de l'informatique et des libertés). In accordance with the Helsinki declaration, written informed consent was obtained from each student before inclusion.

### Population

Out of 630 replies, 147 were incomplete and/or useless. Thus, the final sample consisted of 483 college students (355 women, 128 men), aged between 18 and 24 years (*M*: 20.23; *SD*: 1.99) from the Parisian area (11.6% were not registered at the University Paris Ouest Nanterre La Defense).

### Measures

The questionnaire of the study was divided in two parts. In the first part, students were asked to give socio-demographic data concerning their gender, age, place of residence, current year of studies, study program and university of origin. They also had to report, using a visual analog scale, whether they were satisfied with the discipline they chose at university and whether they had ever repeated an academic year. The second part included six questionnaires:

Self-esteem was measured using the 10-item version of the Rosenberg Self-Esteem Scale (RSES), developed by Rosenberg (1965) and translated and validated in French by Vallieres and his team. All items were answered using a 4-point Likert scale format ranging from strongly agree to strongly disagree (Vallieres and Vallerand, 1990). A score lower than 30 indicates a low self-esteem (Chabrol et al., 2004). Cronbach's alpha coefficient in this study was: (0.86).

Perceived self-efficacy was evaluated using the General Self-Efficacy Scale (GSE), developed by Matthias Jerusalmen and Ralf Schwarse (Luszczynska et al., 2005). It was translated in French and validated by Dumont and his team and consisted of 10 items that are answered using a 4-point Likert scale ranging from “not at all true” to “exactly true” (Dumont et al., 2000). Cronbach's alpha coefficient in this study was: (0.84).

Optimism was assessed using the 10-item revised version of the Life Orientation Test (LOT-R) developed by Scheier and Carver (Trottier et al., 2008). It was translated in French and validated by Sultan and Bureau (Shankland and Martin-Krumm, 2012). A higher score indicates higher optimism. Cronbach's alpha coefficient in this study was: (0.83).

Student's well-being was measured using the Satisfaction With Life Scale (SWLS) developed by Diener et al. (1985); Diener (2006). It was translated in French and validated by Blais and his team and consisted of five items that are answered on a scale from 7 (strongly agree) to 1 (strongly disagree) (Blais et al., 1989). A higher indicates a higher satisfaction. Cronbach's alpha coefficient in this study was: (0.82).

Perceived stress was assessed using the 10-item Perceived Stress Scale (PSS-10). It was developed by Cohen et al. (1983) and translated and validated in French by Bellinghausen et al. (2009). Each item is rated on a 5 points scale from “1” (never) to “5” (very often). It includes two factors: perceived helplessness and perceived self-efficacy (Bellinghausen et al., 2009), and two scores thresholds: a score superior to 24 for anxiety and to 26 for depression (Collange et al., 2013). In this study Cronbach's alpha coefficient in this study was: (0.82).

Psychological distress was evaluated using the 28-item General Health Questionnaire (GHQ-28) scale. This measure was initially described by Goldberg in 1972 (Goldberg and Hillier, 1979) then translated and validated in French by Bolognini et al. (1989). It is divided into 4 subscales which measure somatic symptoms, anxiety/insomnia, social dysfunction and severe depression (Goldberg and Hillier, 1979), each consisting of 7 items that are answered using a 4-point Likert scale (Bolognini et al., 1989). A score greater than or equal to 5 indicates psychological distress (Guelfi, 1993). Cronbach's alpha coefficient in this study was: (0.84).

### Statistical analysis

Statistical analyses were performed using the software Statistica (version 12). First, descriptive analyses (such as percentages, means and standard deviations) were produced. Then, bivariate analyses (Mann–Whitney *U*-test, Spearman's correlation) were performed in order to investigate the possible links between all variables of interests (gender, age, year of studies, UFRclassification at the University of Paris Ouest Nanterre, self-esteem, optimism, generalized self-efficacy and psychological distress (somatic symptoms, anxiety/insomnia, social dysfunction, severe depression). Finally, multivariate analyses (multiple regressions) were run to test the link between perceived stress as the dependent variable and including, as predictors, the year of studies, the academic sector, self-esteem, optimism, the sense of self-efficacy and psychological distress. The significance limit was set at *p* < 0.05.

## Results

### General characteristics of the sample (Table 1)

The final sample consisted of 483 students from the Parisian area (88.41% from the University of Nanterre), of whom the majority were women (73.5%). The average age was 20.23 years (±1.99). Most students lived with their parents (68.7%), did not have kids (88.4%) and reported being somewhat satisfied with their studies (38.5%). This sample included students from all years of studies (first year of license to PhD) and from different academic sectors. The majority of students did not repeat any academic year (77.6%).

**Table 1:** **Participants' sociodemographic and educational characteristics**.

**Participants' characteristics**		**Number (total *N* = 483)**	**Percentage frequency (%)**
Gender	Women	355	73.5
	Men	128	26.5
Residence	Living with parents	332	68.7
	University residence	32	6.6
	Roommate/couple	56	11.6
	Other	63	13
Have children	No	456	94.4
Home university	Paris Nanterre La Defense	427	88.4
Year of studies	L1: First academic year	213	44.1
	L2: Second academic year	109	22.6
	L3: Third academic year	109	22.6
	M1: First year of Master degree	24	4.97
	M2: Second year of Master degree	21	4.4
	PhD	7	1.5
Formation and research units (UFR)	Foreign cultures and languages (LCE)	44	9.1
	Philosophy, information-Communication, Language, Literature, Performing Arts (PHILLIA)	92	19
	Economics, Management, Mathematics, Computer Science (SEGMI)	57	11.8
	Law and Political Science (DSP)	109	22.6
	Psychological Sciences and Educational Sciences (SPSE)	72	14.9
	Social sciences and administration (SSA)	74	15.3
	Other	35	7.3
Repetition of academic year	No	375	77.6
Cursus satisfaction ^*^	Not satisfied at all	14	2.9
	Unsatisfied	30	6.2
	Slightly dissatisfied	84	17.4
	Slightly satisfied	186	38.5
	Satisfied	148	30.6
	Extremely satisfied	21	4.4

* *Level of satisfaction with the university cursus the student picked*.

### Results of the questionnaires used (Table 2)

Low self-esteem was reported by 57.6% of students while 25% of them were slightly satisfied by their well-being and 56.7% had little optimism. The majority of them also reported having a low sense of self-efficacy (62.7%), anxiety (86.3%) and depression (79.3%). Finally, according to their GHQ-28 scores, 72.9% of students declared suffering from psychological distress.

**Table 2:** **Students' results to different questionnaires**.

**Scale**	**Level (threshold)**	**Number (*N* total = 483)**	**Percentage frequency (%)**	**Total**	
				***M***	***SD***
**SWLS:**				22.63	6.28
Life satisfaction	Extremely satisfied (31–35)	35	7.25		
	Satisfied (26–30)	156	32.3		
	Slightly satisfied (21–25)	121	25		
	Neutral (20)	21	4.35		
	Slightly dissatisfied (15–19)	89	18.4		
	Dissatisfied (10–14)	47	9.7		
	Extremely dissatisfied (5–9)	14	2.9		
**RSES:**				28.41	5.43
Self-esteem	Low self-esteem (<30)	278	57.6		
	High self-esteem (≥30)	205	42.44		
**LOT-R:**				12.36	4.77
Optimism	High optimism (19–24)	46	9.5		
	Moderate optimism (14–18)	163	33.75		
	Low optimism (0–13)	274	56.7		
**GSE:**				28.13	4.51
Self-efficacy	Low sense of self-efficacy (<29)	303	62.7		
	High sense of self-efficacy (≥29)	180	37.27		
**PSS-10:**				30.48	6.17
Perceived stress	Anxiety (≥24)	417	86.3		
	Depression (≥26)	383	79.3		
	Perceived helplessness	–	–	19.78	4.42
	Perceived self-efficacy	–	–	10.72	2.53
**GHQ-28:**				8.45	5.32
Psychological distress	Psychological distress (≥5)	352	72.9		
	No psychological distress (<5)	131	27.12		

*M, Mean; SD, standard deviation; SWLS, Satisfaction With Life Scale; RSES, Rosenberg Self-Esteem Scale; LOT-R, Revised version of the Life Orientation Test; GSE, General Self-Efficacy Scale; PSS-10, 10-item Perceived Stress- Scale; GHQ-28, 28-item General Health Questionnaire*.

### Results of gender differences (Table 3)

Results showed that men had a higher sense of self-efficacy than women. The latter had significantly higher scores on perceived stress and both its factors (perceived helplessness and perceived self-efficacy) and presented more psychological distress, including more somatic symptoms and anxiety/insomnia.

**Table 3:** **Results of questionnaires according to gender**.

**Factors**	**Women Mean(*SD*)**	**Men Mean (*SD*)**	***p***
Life satisfaction	22.9(6.27)	21.76 (6.24)	0.05
Self-esteem	28.17(5.44)	28.96 (5.41)	0.23
Optimism	12.21(4.8)	12.76 (4.69)	0.31
General self-efficacy	27.7(4.6)	29.4(4.005)	0.0002^***^
**THE PERCEIVED STRESS**			
Global	31(6.15)	29 (6)	0.002^**^
Perceived helplessness	20(4.42)	18.3 (4.33)	0.02^*^
Perceived self-efficacy	10.95(2.55)	10 (2.38)	0.0001^***^
**PSYCHOLOGICAL DISTRESS**			
Global	8.96 (5.34)	7.03 (5.05)	0.0002^***^
Somatic symptoms	2.8 (1.95)	1.82 (1.68)	0.00000^***^
Anxiety and insomnia	3.3 (2.18)	2.65 (2.16)	0.002^**^
Social dysfunction	1.48 (1.43)	1.29 (1.25)	0.3
Severe depression	1.28 (1.78)	1.25 (1.83)	0.61

SD, standard deviation; p:

* : 0.05.

** :0.01.

*** *:0.001*.

### Results of the links between stress and studied factors (Table 4)

When simultaneously taking into account all studied factors (gender, age, year of studies, UFR classification at the University of Paris Ouest Nanterre, self-esteem, optimism, generalized self-efficacy and psychological distress (somatic symptoms, anxiety/insomnia, social dysfunction, severe depression), we found a significant link between stress and most of these factors. In fact, on the one hand, there was a negative correlation between perceived stress and factors that could be considered as “positive” ones such as self-esteem, optimism and sense of self-efficacy. On the other hand, there was a positive correlation between perceived stress and factors that could be considered as “negative” ones such as psychological distress and its four factors on the GHQ-28 scale including somatic symptoms, anxiety and insomnia, social dysfunction and severe depression.

**Table 4:** **Results of the links between stress and studied factors**.

**Factors**	**Perceived stress**	**Perceived helplessness**	**Perceived self-efficacy**
	***r***	***r***	***r***
Gender	−0.13^**^	−0.1^*^	−0.17^***^
Age	0.07	0.09^*^	−0.02
Year of studies	0.05	0.1^*^	0.06
Formation and research units	0.05	0.003	0.01
Life satisfaction	−0.49^***^	−0.42^***^	−0.45^***^
Self-esteem	−0.58^***^	−0.48^***^	−0.57^***^
Optimism	−0.52^***^	−0.44^***^	−0.48^***^
Self-efficacy	−0.51^***^	−0.38^***^	−0.58^***^
Psychological distress	0.57^***^	0.54^***^	0.45^***^
Somatic symptoms	0.43^***^	0.41^***^	0.34^***^
Anxiety and insomnia	0.50^***^	0.49^***^	0.38^***^
Social dysfunction	0.19^***^	0.18^***^	0.16^**^
Severe depression	0.53^***^	0.49^***^	0.43^***^

r, Spearman's rank correlation coefficients; p:

* : 0.05.

** :0.01.

**** *:0.0001*.

Results showed a significant link between gender and scores on the perceived stress scale, specifically in women who were more stressed on most of the evaluated factors. Analyses did not show any association between perceived stress score (and its factors) and age, year of studies or UFR classification.

Additional studies on perceived helplessness and perceived self-efficacy, two subscales of the PSS-10 scale, showed similar results concerning the negative and positive associations found with perceived stress.

### Results of linear multiple regression analyses (Table 5)

A linear multiple regression analysis was performed using perceived stress as the dependent variable and including as predictors the year of studies, the academic sector, self-esteem, optimism, the sense of self-efficacy and psychological distress. The total variance accounted for by the model was 57% [*F*_(7, 475)_ = 93.269; *p* < 0.0001]. Life satisfaction (Beta = −0.11; *p* = 0.002), self-esteem (Beta = −0.20; *p* = 0.000002), optimism (beta = −0.12; *p* = 0.002), self-efficacy (beta = −0.19; *p* = 0.00) and psychological distress (beta = 0.38; *p* = 0.00) independently and significantly predicted perceived stress. Furthermore, among those variables, self-esteem, optimism and self-efficacy negatively predicted perceived stress and were considered to be “positive” variables while psychological distress positively predicted perceived stress and was considered to be a “negative” variable.

**Table 5:** **Results of linear multiple regression analyses using the PSS-10 score as the dependent variable**.

***N*: 483**	***R* = 76,082,630, *R*^2^ = 57,885,665, *R*^2^ Ajusté = 57,265,033 *F*_(7, 475)_ = *93.269, p < 0.0000, Err-Type de l'Estim.: 4.0347***		
**Factors**	**Beta**	**Standard error of Beta**	***p***
Year of studies	0.03	0.03	0.24
Academic sector	−0.01	0.03	0.57
Life satisfaction	−0.11	0.03	0.002^**^
Self-esteem	−0.20	0.04	0.000002^***^
Optimism	−0.12	0.04	0.002^**^
Self-efficacy	−0.19	0.03	0.00^***^
Psychological distress	0.38	0.03	0.00^***^

p: ^*^: 0.05.

** :0.01.

*** *:0.0001*.

## Discussion

### Prevalence of stress and psychological difficulties evaluated in our french college students sample

In line with several previous research stating the importance of psychological problems among college students (Lafay et al., 2003; Lassarre et al., 2003; Boujut et al., 2009; Strenna et al., 2009), our results show that the students included in our sample (*N* = 483) have high levels of anxiety (86.3%), depression (79.3%), psychological distress (72.9%) and have a low self-esteem (57.6%). The level of stress in our sample is slightly higher than the ones found in the literature(Vandentorren et al., 2005; Strenna et al., 2009; Dachew et al., 2015; Deasy et al., 2015; Larcombe et al., 2016; Weier and Lee, 2016).

### Gender differences

Overall, the psychological difficulties were significantly higher in women than men when it came to perceived stress, perceived helplessness, perceived self-efficacy, global psychological distress, somatic symptoms, anxiety and insomnia. They also have a lower sense of self-efficacy than men. However, we did not find significant differences between women and men concerning self-esteem, life satisfaction, and optimism.

On the one hand, these results are in accordance with many previous research performed in different countries (Backović et al., 2012; Cruz et al., 2013; Shamsuddin et al., 2013) that show that levels of stress and psychological distress are higher in female than male college students (Spitz et al., 2007; Backović et al., 2012; Deasy et al., 2015). On the other hand, our results concerning associations between gender and the sense of self-efficacy are in opposition to other literature data (Follenfant and Meyer, 2003; Ayle and Nagels, 2014). This was also the case for our results concerning the links between gender and self-esteem (Dozot et al., 2009).

### Link between stress and gender, and stress and age, in students

When it comes to the link between stress and gender, our results confirm the significant link previously find in other studies. Female students are usually found to be more stressed than male students (Fornés–Vives et al., 2012; Cruz et al., 2013; Shamsuddin et al., 2013). However, Koochaki et al. (2011) did not find any significant differences according to gender and oppositely, two studies found that male students reported higher stress levels than females (Acharya, 2003; Ahern and Norris, 2011).

When it comes to the link between stress and age, we did not find any associations between these two variables, independently of the studied stress factor. This result is in line with some studies (Koochaki et al., 2011) while others show a negative association between perceived stress and age (Fornés–Vives et al., 2012; Voltmer et al., 2012).

### Stress predictors

Both the year of studies and the academic sector did not have a significant impact on perceived stress in the regression analyses. Nonetheless, and unlike other protective factors (i.e., life satisfaction, self-esteem, optimism and generalized self-efficacy) that negatively predicted perceived stress, we found that psychological distress significantly contributed the most to the variance of perceived stress. The total variance of perceived stress accounted for by the model including all studied factors was 57%. These factors could be considered stress vulnerability factors among students.

Interestingly, previous research found significant associations between perceived stress and: (1) psychological distress (La Rosa et al., 2000; Strenna et al., 2009); (2) self-esteem (Boujut, 2007) optimism (Mazé and Verlhiac, 2013) self-efficacy (Han, 2005). In fact, according to the literature, the most important predictor of stress symptoms in university students was the sense of self-efficacy (Han, 2005). Nevertheless, we found that self-esteem (beta = −0.20; *p* = 0.000002) and self-efficacy (beta = −0.19; *p* = 0.00) negatively predicted it. Regression analysis also showed that psychological distress (beta = 0.38; *p* = 0.00) was the most powerful positive predictor of stress symptoms.

## Potential shortcomings and limitations

This research has a number of limitations. It is limited by a small sample size, which reduced statistical power. Participants mainly consisted of women and were principally recruited from the university of Paris Nanterre La Defense (that only has human sciences' department), which is not representative of all students in the Ile de France region. In addition, our sample was recruited via the internet and this could have limited the participation of more students. Our study also lacked an adequate control group. We used non-randomized sampling. The assessment was solely based on self-reported questionnaires and their results were not validated by a semi-structured interview.

Additionally, the cross-sectional design of our study investigates associations rather than causality. Thus, future research needs to replicate these findings using a longitudinal design to compare students' states at the start vs. the end of their university year.

## Conclusion

Most university students included in this study displayed high levels of perceived stress and psychological distress and low levels of self-esteem, optimism and self-efficacy. The multivariate model included in our research helped us identify the most important stress-vulnerability factors that should be taken into consideration when identifying stress among students and when establishing prevention and intervention programs.

In fact, these findings suggest that focusing on the sense of self-efficacy and self-esteem could be essential in intervention programs for students.

Future research could benefit from including more homogeneous samples regarding gender and from recruiting students from a larger variety of academic sectors (medicine, physics, etc.).

## Author contributions

DS: Conception or design of the work, data collection, data analysis and interpretation, drafting the article, final approval of the version to be published. NC: Conception or design of the work, data collection, critical revision of the article, final approval of the version to be published. LR: Conception or design of the work data analysis and interpretation critical revision of the article, final approval of the version to be published.

### Conflict of interest statement

The authors declare that the research was conducted in the absence of any commercial or financial relationships that could be construed as a potential conflict of interest.
